# Temperature-sensitive mosquito TRP channel rescues touch deficits caused by knock-out of a DEG/ENaC channel in *C. elegans* glia

**DOI:** 10.17912/micropub.biology.000209

**Published:** 2020-01-02

**Authors:** Ying Wang, Laura Bianchi

**Affiliations:** 1 Department of Physiology and Biophysics, University of Miami

**Figure 1.**
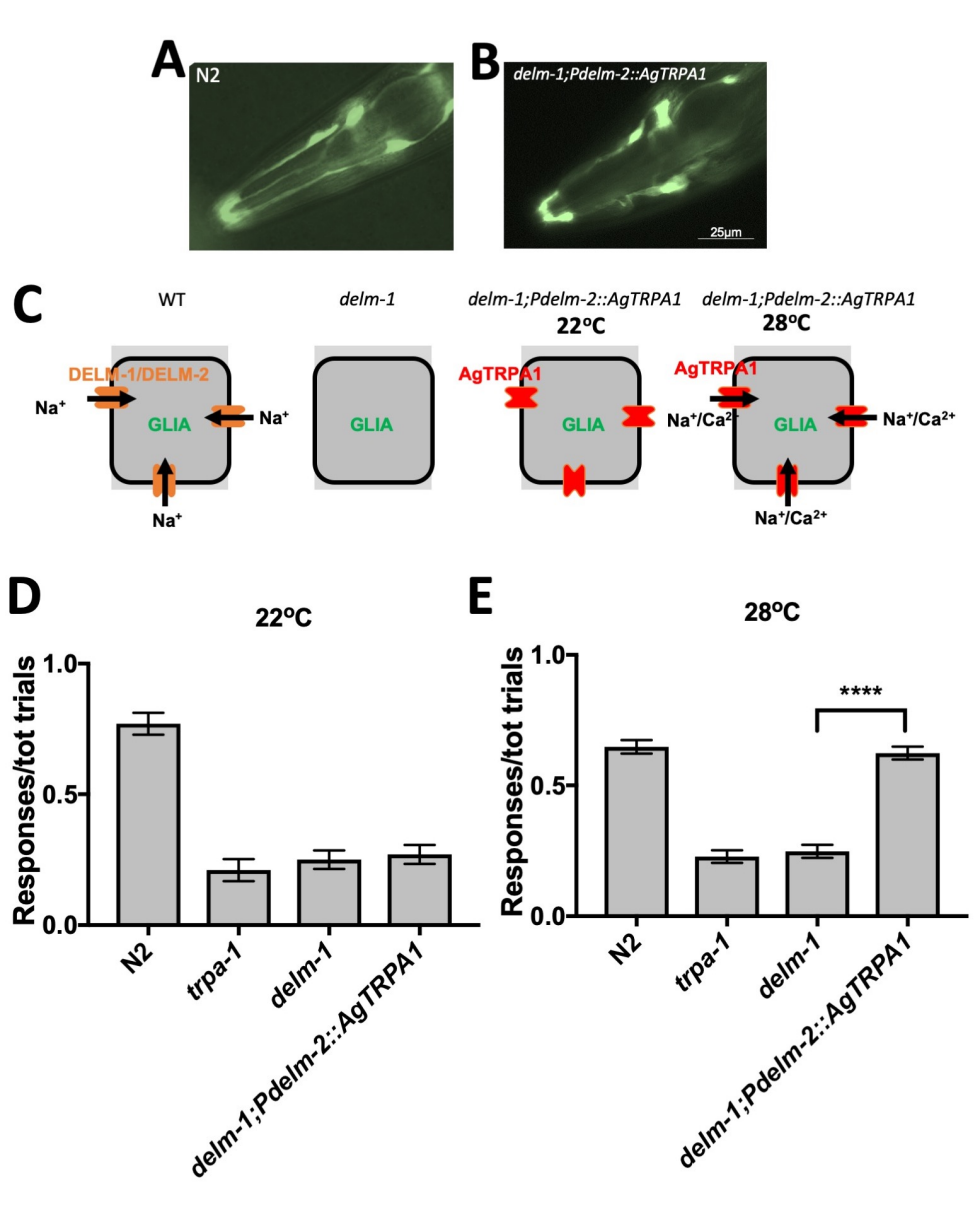
(A)Photograph of the head of a transgenic animal expressing GFP under the control the promoter of *delm-2*(*Pdelm-2::GFP*) (front is to the left). (B) same as in (A) for a *delm-1* mutant expressing AgTRPA1 under the control of *Pdelm-2* (*delm-1;Pdelm-2::AgTRPA1;Pdelm-2::GFP*). *delm-2* is expressed in OLQ and IL socket glia (Han et al., 2013). (C) Schematic representation of a OLQ or IL socket glia in the indicated genetic backgrounds. In animals expressing AgTRPA1 in socket glia, influx of cations is present only at temperatures above 27^o^C due to the temperature sensitivity of AgTRPA1. WT is N2. (D) Ratios of responses (responses/total number of trials, labelled on the Y axis as “responses/tot trials”) assayed at 22˚C for wild type animals (N2, 0.770 ± 0.042, n = 20), *trpa-1(ok999)* (0.210 ± 0.042, n = 20), *delm-1(ok1226)* (0.250 ± 0.035, n = 20), and *delm-1* expressing mosquito TRPA1 (AgTRPA1) in OLQ and IL1 socket glia (*delm-1(ok1226);Pdelm-2::AgTRPA1*, 0.270 ± 0.036, n = 20). (E) Ratios of responses assayed at 28˚C for wild type animals (N2, 0.6480 ± 0.026, n = 50), *trpa-1(ok999)* (0.228 ± 0.024, n = 50), *delm-1(ok1226)* (0.248 ± 0.025, n = 50), and *delm-1* expressing mosquito TRPA1 (AgTRPA1) in OLQ and IL1 socket glia (*delm-1(ok1226);Pdelm-2::AgTRPA1*, 0.624 ± 0.025, n = 50). Data are expressed as means ± SE, ****p<0.0001 by ANOVA with Bonferroni correction.

## Description

Recent studies have shown that accessory cells of mammalian touch receptors, such as the Merkel receptors, play an essential role in transducing touch (Pawson et al., 2009, Maricich et al., 2009, Maksimovic et al., 2014, Woo et al., 2014, Ikeda et al., 2014, Woo et al., 2015). Similarly, in *C. elegans*, we found that OLQ and IL glial socket cells are needed for responses to nose touch. More specifically, we reported that these glial cells express DEG/ENaC Na^+^ channel DELM-1 and DELM-2, which are needed specifically in glia for OLQ neuronal responses to touch and nose-touch avoidance behavior. We also showed that nose touch responses are restored by overexpression of the worm inward rectifier K^+^ channel IRK-2 in socket glia of *delm-1* mutants (Han et al., 2013). These published results suggest that glial Na^+^ and K^+^ channels are needed for the function of the associated touch receptor neurons.

We next wondered whether it is the influx of cations or expression of DEG/ENaC Na^+^channels that is needed in glia for nose touch avoidance. To distinguish between these possibilities, we expressed the temperature-sensitive mosquito TRPA1, AgTRPA1, in socket glia of *delm-1* mutants and assayed nose-touch avoidance (Kang et al., 2012). AgTRPA1 belongs to the TRP family of ion channels and is a Na^+^ and Ca^2+^ permeable channel (Kang et al., 2012). Furthermore, AgTRPA1 has the advantage that it is activated by temperature, which allows for a direct test of whether the influx of cations in glia is needed for nose touch response.

When we expressed AgTRPA1 in OLQ and IL socket glia of *delm-1* mutants, we did not find any gross abnormalities of glial cells (Fig. 1 A and B). We previously published that knock-out of *delm-1* does not alter glial morphology either (Han et al., 2013). When we assayed nose touch at 22˚C, when AgTRPA1 channel is closed, we found no rescue of the nose touch response (Fig. 1 C and D). On the contrary, when assayed at 28˚C, when AgTRPA1 is open, the transgenic animals expressing AgTRPA1 in OLQ and IL socket glia of *delm-1* mutants, showed restoration of nose touch sensitivity (Fig. 1 C and E). These results support that movement of cations across the OLQ and IL socket glial plasma membrane is needed for nose touch response in *C. elegans*, suggesting that depolarization of the glial plasma membrane is what is required for nose touch avoidance behavior. Given that we published that overexpression of worm IRK-2 restores nose touch defects of *delm-1* mutants, we propose that depolarization of the glial plasma membrane might be required for vectorial transport of K^+^ from the surrounding tissue to the microenvironment between glia and neurons to regulate neuronal output (Wang et al., 2008, Wang et al., 2012), as suggested by work in Pacinian corpuscles (Ilyinsky et al., 1976).

## Methods

**Fluorescence microscopy:** Animals were immobilized on 2% agarose pads using 20 mM NaN_3_. Images were obtained using an Evos FL Auto 2 Imaging System (Invitrogen), equipped with a 40x objective (Olympus), and accompanying Evos FL Auto 2 software. Black and white images were artificially colored green.

**Behavioral assays:** Young adult animals were placed on plates seeded with a small and thin lawn of OP50 and allowed 30 minutes for recovery. An eyelash hair was placed in front of a forward-moving animal such that the animal encountered the eyelash perpendicular to its nose. A response was recorded if the animal reversed or moved the head away upon contacting the eyelash. Ten animals per strain were tested five times each with 30 seconds intervals in between touches. Data are displayed as responses given by each animal over the course of the five touches, with means obtained by averaging the response ratios from different animals.

**Molecular biology:** To drive expression of mosquito TRPA1 (AgTRPA1) in the OLQ and IL socket glia, we swapped the GFP sequence in the ppD95.75 vector containing the promoter of *delm-2* with AgTRPA1 (Han et al., 2013).

## Reagents

***C. elegans* growth and maintenance:** Animals were grown on standard nematode growth medium (NGM) seeded with *Escherichia coli* strain OP50 and maintained at 20˚C. Experiments were performed on young adult hermaphrodites.

***C. elegans* strains:** Nematode strains used were as follows: N2 wild type Bristol, RB1052 *trpa-1(ok999) IV*, BLC53 *blcEx38* *[Pdelm-1::RFP Pdelm-2::GFP]*, RB1177 *delm-1(ok1226) IV* (Han et al., 2013), and BLC263 *delm-1(ok1226) IV; blcEx104 [Pdelm-2::AgTRPA1 Pdelm-2::GFP].* Germline transformation was carried out as described previously (Mello et al., 1991); DNA was micro-injected in the gonads of young adults and transgenic animals were selected and propagated based on GFP and/or RFP fluorescence in the OLQ and IL socket glia.
